# Round the Hospitals

**Published:** 1917-11-10

**Authors:** 


### ROUND THE HOSPITALS.

MISS RUNDLE, the secretary of the College of Nursing, at the invitation of Miss Wallace, R.R.C., matron, attended a meeting at the Southwark Military Hospital, Dulwich, on November 5, the Rev. Howard Nixon, chaplain, in the chair. The meeting was well attended and full of interest. It demonstrated the wisdom of the British Women's Hospital Committee in putting the educational side of the College, its foundation and endowment, prominently forward. The discussion which followed Miss Rundle's address proved the great interest nurses take in the educational scheme of the College, and there is no portion of the programme of the College of Nursing which appeals more strongly to the sisters and nurses throughout the country. Other topics of interest were the future standards and methods of conducting examinations, problems which the secretary reminded the meeting could not be settled now, but by the members of the College through their own elected representatives, the first batch of whom will be appointed, in accordance with the constitution, by the election of one-third of the members of the Council of the College in March next. This fact really limits the time which is open for criticism by the enemy to the effect that the existing College is

**ROUND THE HOSPITALS**—(*continued*).

non-effective. It further demonstrates the prejudiced nature of such criticism which ignores the constitution of the College, which provides that the whole of the members of the College Council are subject to election by the members, and that this change will proceed in due order continuously from March 1918.

We publish on another page a letter from "Ierne" criticising the action of the College Board in Ireland because it has not dealt exhaustively with the objects and every branch of the work which the College was established to undertake, organise, and perfect. It is fair to presume that "Ierne" was once a baby, like the College Board, and would be the first to resent a criticism of her mother's policy in regard to herself, because all her teeth had not been cut within twelve months of her birth. The truth is that the College has steadily worked, despite the handicap it has experienced in the avoidable delays which have been due to a want of business aptitude and apprehension on the part of those responsible for the policy and decisions of the Council of the Royal British Nurses' Association. Now this cause for delay may be regarded as at an end, the Council is free to pursue an active propaganda, involving the full development of the educational scheme of the College and the provision of the necessary funds to place it once for all on an adequate financial basis. Such a policy must secure for British nurses a position and standing in the world which should be unequalled by that open to the nurses of any other nation.

The discussion at the Southwark meeting on the 5th instant shows how welcome this new position is to sisters and nurses generally, and we welcome this evidence that the members of the profession individually, apart from all organisations of every kind, are awakening, and have the keen intelligence and business gifts necessary to eliminate the wheat from the chaff. This should unite them behind the College in their own best interests and for their future uplifting. This awakening may be taken as a proof that the democratic basis on which the College of Nursing rests, and its scheme to make the nurses the controllers of their own advancement and destiny, is at length being understood and appreciated to an extent which must be continuously increased by the reports of the proceedings of the meetings of the College Council, and the growth of its Register by the election of certificated nurses who have completed their training at recognised schools in England, Wales, Scotland, and Ireland. An encouraging proof of this is the list of nurses accepted for membership of the College by the Council at its last meeting, which we have the pleasure to publish this week. This list includes nurses trained at ten Metropolitan training-schools and three London Poor-Law infirmaries, twelve provincial training-schools, and eight provincial Poor-Law infirmaries. It further includes nurses trained at ten Scottish training-schools attached to important hospitals, and four Scottish Poor-Law institutions. The Irish Board at its last meeting admitted to the Register nurses trained at eight important Irish hospitals and one provincial Irish Poor-Law infirmary.

On page 101 of The Hospital for November 3 we published portions of Miss Matheson's address to the meeting in Dublin of the Irish Branch of the College of Nursing on October 29. Dr. Peacocke, the chairman of the Irish Board, properly insisted that the Irish Branch of the College was fully representative of Ireland. The members of its Council numbered doctors and nurses from all parts of the country, including Belfast, Limerick, Cork, Galway, and Waterford. Dr. Peacocke expressed his keen interest in the work of the College, believing it to be the organisation which would do the most for nurses. In the discussion which followed, Sir John William Moore, replying to a statement of Miss Kearns to the effect that Miss Matheson had made the statement that Irish training was inferior to British training, said, "It is admitted on all sides that the training in Ireland is unequal, and that reform is needed." There was no question in Miss Matheson's letter of English training at all. The inequality was in Ireland in the different standards of the various training-schools. No statement had been made as to the relative value of English and Irish training. In reply to a question from Miss Violet Roberts asking on what she rested her statement that the Irish Nursing Board was based on politics, Miss Matheson pointed out that she had made no such assertion. Her point was to beg Irish nurses not to allow political considerations to interfere with or prejudice their decision as to the future of their profession. In Ireland political considerations came into every question which had any connection whatsoever with England. What Miss Matheson had said was quite independent of the Irish Nursing Board. Replying to further questions, Miss Matheson announced that the examinations for the College would be carried out in Ireland by Irish examiners. Speaking of the V.A.D.s she said that even those with two or three years' experience at the Front would not be placed on the College Register unless they took the same training and passed the same examinations as the trained nurse. Whatever modifications might be found feasible, it was certainly not the intention of the College to place V.A.D.s on the General Register until they had taken their general training and passed the examination for a trained nurse.

The British Red Cross Society, Scottish Branch, City of Edinburgh Committee, sends us a list containing twenty-four names of matrons and nurses appointed to positions in hospitals both at home and abroad since July 6 last. They state that the first line auxiliary hospitals in the Edinburgh district are still making demands on their nursing resources. The committee will be glad to receive applications from trained nurses, more especially from those who can fill responsible posts as matrons or sisters

**ROUND THE HOSPITALS—(*continued*).**

in charge, addressed to Miss Ethel Maxtone Graham, 80A Princes Street, Edinburgh. The names received can then be added to the British Red Cross Society's Register, to be called up as occasion demands.

---

THE Liverpool Select Vestry, which is notorious for its public utterances and remarkable for its administration of that ancient incubus upon Liverpool as a city, the Brownlow Hill Institution, has surpassed itself by adopting a resolution strongly urging the Local Government Board to accord to the matron of Brownlow Hill, her assistants and nurses, and to the nursing staffs of Poor-Law and civilian hospitals generally, official appreciation of the extremely valuable services which they, working since the beginning of the war with greatly depleted forces, and in the face of enormous difficulties, have rendered to the community by their unostentatious and self-sacrificing ministrations to the sick poor. It was pointed out that while honours had been given by the War Office to matrons and nurses of the new military hospitals working under hygienic conditions, the nursing staffs remaining in the older hospitals and over-worked, under less happy arrangements, were without due recognition. This would seem to make this claim rest upon the great sympathy due to the Brownlow Hill nurses and other Poor-Law nursing staffs where hygienic conditions do not prevail, a disgraceful condition of affairs where it exists, which appears to call for immediate and forcible action on the part of the Local Government Board to enforce efficiency upon those who may be responsible for any such neglect of a plain public duty. Some speakers appeared to be under the impression that honours are apt to be distributed by placing all the available names in a hat and drawing therefrom a specified percentage of the hat's contents.

---

ON the 14th instant H.R.H. Princess Patricia is to open, at the Central Hall, Westminster, at 3 P.M., the Englishwoman Exhibition of Arts and Handicrafts. The exhibition is to remain open until Saturday, the 24th instant, from 11 A.M. to 6 P.M. daily. 1917 is the seventh year that this exhibition has been held, and it has claimed to have given a valuable stimulus to home industries. Upwards of fifty industries will be represented by well-known craft workers, and the exhibits will include weaving, pottery, glasswork, toys, model buildings, boats and aeroplanes, with hospital requisites, and a model of the Royal Free Hospital. There is to be an exhibit by sailors of the 1st Naval Division interned in Holland.

---

THE Bishop of London, speaking at the Diamond Jubilee meeting of the Ranyard Nurses, testified to the high regard in which these nurses were held by those amongst whom they laboured. "Even in this time of air-raids, if a Ranyard nurse tells the people that the Zeppelins are not likely to come to-night, they have such confidence in her that they will go quietly home, and if there is a raid, why then they gather together where she tells them, and she holds a little prayer meeting and calms them with her words, without ever a thought for her own comfort." The Bishop added: "Think of all that these nurses do towards preserving the lives of the babies. Why, even I, a mere man, and a bachelor at that, even I know that beefsteak and gin are not the best kind of diet for a baby of three months old. But some of the mothers need to be helped in things of this kind, and the Ranyard nurses are just the people to do it." We have known the work of the Ranyard Nurses' organisation and welcomed it continuously practically throughout the whole of its existence. It is one which everyone at this time should cheerfully support by sending something in aid. Donations will be gratefully received by the Secretary, Ranyard House, 25 Russell Square, London, W.C. 1.

---

FORMED in 1903, the Nurses' Missionary League aims at uniting nurses together for mutual help in the spiritual life, and to bring before them the great need for missionary workers in the foreign field. Beginning with four London hospitals the League has extended its ramifications to the Provinces, and the annual meeting which was held on the 31st ult. at the University Hall, Golden Square, proves the accuracy of the claim that this League continues to increase in spite of the strain of war work. It is stated that members of the Nurses' Missionary League may be found in every branch of military nursing. Dr. Catherine Ironside, from Persia, gave several instances of the methods adopted to induce rich Persian ladies to undergo operations in the hospital at Ispahan. In the case of a very bigoted Mahomedan private patient who was dressed daily by a lady doctor with "no opportunity for religious conversation"! when Sunday came the Mahomedan patient remarked, "I thought this was the Christian's rest day," and got the reply that necessary work to relieve suffering must be done on Sundays. Here is the patient's answer, "Yours must be a very good religion. Tell me more about it." So the mission progresses in the East. The Rev. H. S. B. Holland, in his address, maintained that unity was God's great plan for all nations, but man had failed through aiming at lesser goals such as freedom. This remarkable statement requires explanation, for as it stands it would seem to put aside Christ's teaching, and deny the teachings of humanity which demonstrate that freedom is an essential attribute to the unfettered and devout worship of Almighty God by mankind.

---

WE are indebted to Miss Cable, the matron, whose own room is one of the most interesting and helpful sanctums in British hospitals, for the following interesting report: At Salisbury Infirmary jam-making and pickling are looked upon as part of the summer work. The autumn brings vegetable and apple storing, as large quantities are received from Harvest Thanksgiving Services. This year

**ROUND THE HOSPITALS**—(*continued*).

about 5 cwt. of jam has been made, and a considerable amount of pickle. Eggs were preserved during the weeks when prices were at the lowest, and they are proving very valuable now. Potatoes, root vegetables, and apples in great quantities have been stored in outhouses, and even with a full hospital will last probably until Christmas. With the limited staff the work this year has been unduly heavy, but as practically every member took some part in fruit-gathering, or picking, or vegetable storing the work has been satisfactorily accomplished, and the stores must effect considerable saving in these expensive days.

Miss F. A. Goodacre, assistant matron of the Royal Infirmary, Preston, reports that it has been the invariable practice of Miss Marks, the matron, herself to make all the jam and marmalade required for the use of the hospital. It is a matter of pride with the matron to see that as many home-made articles of diet as possible shall be provided, as this practice secures a maximum of variety in food. Last year 1,270 lb. of fruit were made into jam, but this season the amount had to be reduced to one-third, owing to the shortage of sugar. A sufficient quantity of pickles of various kinds for the year has been possible, and chutney is to follow. Two thousand eggs were preserved while they were at their cheapest in the spring and early summer. In addition 250 lb. of fruit have been successfully preserved in ordinary glass jam-jars. The following is the recipe adopted: Choose sound, dry fruit, not too ripe; carefully pick over, wipe, and pack into clean glass jars or wide-necked bottles. Place in a moderate oven, leaving door open until jars are warm to prevent their cracking. Close door and leave until fruit cracks, usually about twenty minutes. Have in readiness a plentiful supply of boiling water. Take jars out of the oven one at a time, fill with boiling water, and immediately seal carefully with parchment paper to exclude all air. Fruit preserved in this way has proved to be popular from the power it confers on the matron to give continuous variety in the menu for every table which has to be provided.

A welfare supervisor, engaged in some large works which have a dental department, expresses satisfaction based on experience that many factories are now including dental treatment for their employees as a recognised part of the works welfare department. The importance of careful and regular attention to the teeth as essential to the health and efficiency of a worker is now becoming generally realised. Some large works, including those of Reckitts' Blue, for instance, actually employ a whole-time dentist, who remains on the premises and is in daily attendance on the workers. This plan ensures the best work and better results in the greater establishments. Other employers find benefit from their point of view when they arrange for a dentist to pay periodical visits under an agreement which provides special treatment for each worker, and recognises weekly instalments of the fee for the treatment received. Women workers are found everywhere. Their advent, as the present instance demonstrates, has proved on the whole an unmixed blessing, and we congratulate Messrs. Reckitts and other large employers of labour, who have taken up the question of teeth and provided the means to secure full benefits to their employees from the organised treatment provided for them.

☛ It is our invariable practice to pay for all items of news which we may publish. The minimum payment is 5s. Paragraphs of about twenty lines in length—that is to say, of 150 words or less—are preferred. Contributions should be addressed to The Editor, The Hospital, 28 and 29 Southampton Street, Strand, London, W.C. 2., and, to be in time for the current week's issue, should reach him by the first post on Mondays.

Scottish Training-schools.

Royal Infirmary, Edinburgh; Royal Infirmary, Glasgow; Western Infirmary, Glasgow; Victoria Infirmary, Glasgow; Royal Infirmary, Aberdeen; Royal Infirmary, Dundee; Royal Alexandra Infirmary, Paisley; Northern Infirmary, Inverness; Kilmarnock Infirmary; Deaconess Hospital, Edinburgh; Eastern District Hospital, Glasgow; Western District Hospital, Glasgow; Stobhill Hospital, Glasgow; Merryflatts Hospital, Govan.

Irish Training-schools.

